# Influence of Plate Design, Thickness, and Fixation Architecture on Mandibular Advancement Stability: A Finite Element Analysis

**DOI:** 10.3390/jcm15041436

**Published:** 2026-02-12

**Authors:** Sergio Olate, Víctor Ravelo, Henry García Guevara, Roberto Sacco, Marcelo Parra, Marcio de Moraes

**Affiliations:** 1 Department of Oral Diagnosis, Division of Oral and Maxillofacial Surgery, Faculty of Dentistry, State University of Campinas, Piracicaba 13414-018, SP, Brazil; sergio.olate@ufrontera.cl (S.O.); marciom@unicamp.br (M.d.M.); 2 Center of Excellence in Morphological and Surgical Studies (CEMyQ), Universidad de La Frontera, Temuco 4811230, Chile; 3 Doctoral Program in Morphological Sciences, Universidad de La Frontera, Temuco 4811230, Chile; victor.ravelo.s@gmail.com; 4 Facultad Ciencias de la Salud, Universidad Autónoma de Chile, Temuco 4810101, Chile; 5 Division for Oral and Maxillofacial Surgery, Hospital Ortopedico Infantil, Caracas 1060, Venezuela; henryagg@gmail.com; 6 Department of Oral Surgery, La Floresta Medical Institute, Caracas 1060, Venezuela; 7 Department of Oral Surgery, Faculty of Dentistry, Oral and Craniofacial Sciences, King’s College London, London SE1 9RT, UK; roberto.sacco@manchester.ac.uk; 8 Department of Adult Dentistry, Faculty of Dentistry, Universidad de La Frontera, Temuco 4811230, Chile

**Keywords:** orthognathic surgery, internal rigid fixation, plate

## Abstract

**Background**: Mandibular advancement is a commonly performed surgical procedure for the treatment of mandibular retrognathia and Class II dentofacial deformities; however, large advancements impose increased mechanical demands on fixation systems. Despite the availability of various fixation strategies, standard straight plate systems remain widely used worldwide due to their availability, cost-effectiveness, and clinical familiarity. Continuous biomechanical evaluation of these systems is therefore required to optimize stability and performance under demanding conditions. **Objectives**: The aim of this study was to evaluate the influence of plate design, plate thickness, and fixation architecture on the mechanical stability of mandibular advancement using finite element analysis. **Methods**: A three-dimensional finite element model simulating a unilateral mandibular osteotomy with a 10 mm gap was generated as mandibular advancement was developed. Fifteen fixation configurations were analyzed, including variations in plate design (simple and reinforced plates with partial or total inferior mesh extension), plate thickness (0.8 mm and 1.0 mm), and fixation architecture using independent plate systems (LN) or integrated fixation systems (FM). A vertical load was applied to the lower central incisor to simulate functional loading. Outcome measures included global equivalent stress considering screws and plate, equivalent stress within the plate, and global deformation of the fixation system. **Results**: The analyses demonstrated distinct mechanical behaviors among the evaluated configurations. Differences in stress distribution and deformation were observed according to plate design, thickness, and fixation architecture. Reinforced designs, increased plate thickness, and integrated fixation systems showed reduced deformation and more favorable stress distribution when compared with simple plate configurations. **Conclusions**: Plate design, thickness, and fixation architecture influenced the mechanical stability of mandibular advancement, supporting the importance of biomechanical optimization of standard fixation systems, particularly in large mandibular advancements.

## 1. Introduction

Although favorable clinical outcomes are commonly reported in large mandibular advancement, the mechanical stability of fixation systems remains a critical determinant of early postoperative behavior. This aspect becomes particularly relevant in cases involving large mandibular advancements, where increased functional loads and bending moments challenge the ability of fixation devices to maintain stable segment positioning [1].

From a technical perspective, distraction osteogenesis is a well-established surgical approach for mandibular advancement in patients with mandibular retrognathia and Class II dentofacial deformities, providing favorable outcomes for both patients and surgeons [2]. Nevertheless, internal rigid fixation remains the most employed technique in mandibular advancement and reconstructive procedures.

Rigid internal fixation using plates and screws is the most frequently employed method for bilateral sagittal split osteotomy [3]. Over time, numerous fixation strategies have been proposed, including variations in plate geometry, thickness, number of plates, and fixation configuration [4]. Standard straight plates continue to be widely used worldwide, largely due to their availability, cost-effectiveness, and surgical familiarity, even in clinically demanding scenarios [3,4].

Finite element analysis (FEA) has become an established and valuable tool for evaluating the biomechanical behavior of osteosynthesis systems under controlled conditions. This approach allows detailed assessment of stress distribution and deformation patterns within fixation devices and supporting structures, providing insights that are difficult to obtain through clinical studies alone [5].

The need for further mechanical investigations persists. Many previously published studies focus on isolated variables or a limited number of fixation configurations, and systematic evaluations addressing the combined influence of plate design, thickness, and fixation architecture remain limited. In addition, the continuous development of modified plate designs and fixation concepts reinforces the importance of ongoing biomechanical assessment to better understand their mechanical performance [6].

Exploration of alternative plate designs and fixation architectures is particularly relevant when aiming to improve stability in challenging clinical situations. However, the mechanical benefits of these modifications must be critically evaluated to determine whether increased structural complexity results in meaningful improvements in stability and predictability [7].

At the same time, the increasing availability of patient-specific and customized fixation systems has introduced new options for mandibular stabilization. While such systems may offer improved anatomical adaptation, they are frequently associated with higher costs, longer production times, and limited accessibility [8,9]. As a result, standard fixation systems remain the primary option in most clinical settings worldwide, highlighting the importance of optimizing their design and application [9].

In the context of large mandibular advancements [10], where mechanical demands are particularly high, a clear understanding of how design-related variables influence fixation performance is essential. Evaluating the mechanical behavior of commonly used standard fixation systems may provide clinically relevant information to support evidence-based decision-making and assist clinicians in selecting the most appropriate fixation strategy according to specific surgical requirements.

By comparing multiple configurations of standard straight plate systems using finite element analysis, the study characterizes stress distribution and deformation patterns to determine how plate design, plate thickness, and fixation architecture influence mechanical stability and identify the strategies that provide the greatest mechanical performance. Therefore, the aim of the present study was to systematically evaluate the influence of plate design, plate thickness, and fixation architecture on the mechanical stability of mandibular advancement using finite element analysis.

## 2. Materials and Methods

***Study design and surgical simulation***

A biomechanical study based on finite element analysis (FEA) was conducted to evaluate the mechanical behavior of different mandibular fixation plate systems under simulated mandibular advancement. The aim was to compare the structural performance of straight plates with different designs, thicknesses, and fixation architectures under standardized loading conditions.

This study did not require Ethics Committee or Institutional Review Board approval because of the nature of data; it does not involve human participants, animals, biological samples, or identifiable patient data.

A three-dimensional mandibular model was generated to simulate a unilateral mandibular osteotomy associated with mandibular advancement. A 10 mm mandibular advancement was applied in all simulations, as this magnitude is frequently reported for the correction of severe deformities of the mandible and represents a clinically relevant condition. This degree of displacement additionally represents a biomechanically demanding scenario for fixation systems.

Boundary conditions were defined by constraining the bilateral condylar regions to prevent rigid body motion. A static axial load of 61 N was applied at the level of the central incisors to simulate anterior bite loading [1]. The 10 mm gap was introduced at the osteotomy site to represent a worst-case discontinuity and evaluate fixation stability, the contact between fixation hardware and bone was modeled as bonded, while the osteotomy surfaces were defined without contact interaction [11]. The mandible and fixation system were assumed to be homogeneous, isotropic, and linearly elastic materials. Screws were simplified as smooth cylinders without explicit threads. This model was designed to allow comparative mechanical evaluation of different fixation configurations rather than to replicate full in vivo mandibular biomechanics.

The designs of the titanium plate and screw models were performed by *Evolve Implantes* (São Caetano do Sul, SP, Brazil) then imported into the CAE (computer-aided engineering) environment using ANSYS^®^ Workbench™ 12 (Ansys Inc., Canonsburg, PA, USA, EE.UU) for the finite element analysis.

For all models, a single vertical load was applied at the mandibular central incisor, simulating an anterior functional load that induces flexural forces on the distal mandibular segment and the fixation system.

The gap was intentionally introduced at the osteotomy site to eliminate any potential load transfer through bone contact or interfragmentary friction. This configuration forces the entire axial load to be transmitted exclusively through the fixation system, allowing the plates and screws to behave as a true bridging structure. This worst-case mechanical scenario amplifies differences between fixation designs and enables a direct assessment of the structural performance of each system, independent of biological support from bone contact. Therefore, the model was conceived to evaluate the intrinsic mechanical behavior of the fixation constructs rather than to replicate clinical osteotomy conditions.

Boundary conditions were kept identical across all simulations to ensure direct comparability between fixation configurations.

A total of 15 fixation configurations were analyzed, categorized according to plate design, plate thickness, and fixation architecture.

***Plate design: Four straight plate designs were evaluated:***

- Simple straight plate.
- Straight plate with reinforcement near the osteotomy site.
- Reinforced straight plate with partial inferior metallic mesh extension.
- Reinforced straight plate with full inferior metallic mesh extension.

***Plate thickness:***

- 0.8 mm thickness: tests 1–11.
- 1.0 mm thickness: tests 12–15.

***Fixation architecture:***

Two fixation architectures were assessed. Configurations included single-plate systems, dual LN plates, combined plate designs, and FM systems with reinforcement and inferior mesh extension.

- LN (Linear model): Individual plates or combinations of independent plates.
- FM (Frame model): Integrated system consisting of superior and inferior plates connected by a metallic arm, forming a unified structural frame.

***Outcome variables***

**Figure 1:**
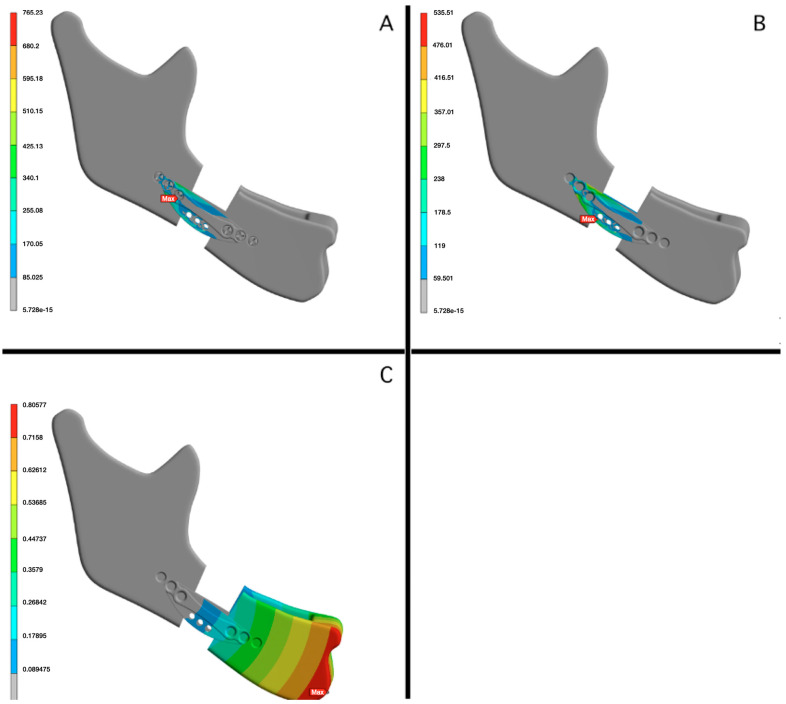
Loading conditions applied in the finite element models: Representative three-dimensional finite element models illustrating the loading conditions applied in the study. (**A**) Mandibular advancement model simulating a 10 mm gap advancement following mandibular osteotomy, with fixation systems positioned across the osteotomy line. (**B**) Boundary conditions showing mandibular constraints applied at the condylar regions to simulate joint stabilization during loading. (**C**) Application of a vertical occlusal load at the lower central incisor, representing a standardized functional loading condition used for all simulations.

Three mechanical variables were recorded for each simulation (Figure 1):

1. Global equivalent stress including screws and plate (A, MPa): maximum equivalent stress considering both screws and plate components.
2. Equivalent stress in the plate (B, MPa): maximum equivalent stress recorded exclusively in the fixation plate.
3. Global deformation of the fixation system (C, mm): maximum displacement of the fixation system, used as an indicator of structural stability under load.

These outcome variables were selected to assess load distribution, implant mechanical demand, and primary stability of the fixation systems. Results were analyzed descriptively and comparatively, focusing on: the effect of plate design, the number of plates used, LN versus FM fixation architecture, and the influence of plate thickness (0.8 mm vs. 1.0 mm).

## 3. Results

Finite element analysis was used as a comparative biomechanical tool to evaluate relative mechanical behavior under a static loading condition. The results reflect the response of the fixation systems under the defined simulation parameters and should be interpreted within the inherent limitations of FEA.

A total of 15 finite element simulations were performed to evaluate the mechanical behavior of different mandibular fixation systems following a 10 mm mandibular gap (Table 1). The fixation configurations varied according to plate design, number of plates, fixation architecture (LN vs. FM), and plate thickness (0.8 mm and 1.0 mm). Three outcome variables were analyzed in all simulations: global equivalent stress including screws and plate (A), equivalent stress in the plate (B), and global deformation of the fixation system (C).

***Overall mechanical behavior***

A wide range of mechanical responses was observed among the fixation configurations. Global equivalent stress values (A) averaged 435.47 ± 205.04 MPa, with a range from 360.24 MPa to 931.98 MPa, whereas equivalent stress in the plate (B) showed a mean value of 356.29 ± 194.04 MPa, ranging between 246.72 MPa and 809.38 MPa. The global deformation of the fixation system (C) varied markedly, with values ranging from 2.7773 mm in the least stable configurations to 0.25263 mm in the most stable systems.

Single LN plate configurations with 0.8 mm thickness and without reinforcement (tests 1 and 2) showed the highest deformation values (2.7773 mm and 2.7643 mm, respectively), associated with high global and plate stress values exceeding 800 MPa. These configurations demonstrated the lowest structural rigidity under the applied load.

***Effect of plate design and reinforcement***

Progressive modification of plate design resulted in a consistent reduction in system deformation. LN plates with reinforcement and partial inferior mesh extension (test 3) reduced deformation to 1.1526 mm, while the addition of a total inferior mesh extension (test 4) further reduced deformation to 0.80527 mm. These changes were accompanied by a reduction in equivalent plate stress from values above 800 MPa to 535.51 MPa.

Combined plate configurations using a simple LN plate with a reinforced LN plate showed further reductions in deformation. Test 7 exhibited a deformation of 0.54105 mm, while the configuration including a reinforced plate with total mesh extension (test 8) reduced deformation to 0.34384 mm, with corresponding global and plate stress values below 560 MPa.

***Single-plate versus dual-plate LN configurations***

Dual LN plate configurations with 0.8 mm thickness (tests 5 and 6) demonstrated a substantial reduction in deformation compared with single-plate systems, with deformation values of 0.63842 mm and 0.63641 mm, respectively. These configurations also exhibited lower global stress values (394.06 MPa and 388.45 MPa) and plate stress values between 331.6 MPa and 360.37 MPa.

***Comparison between LN and FM fixation architectures***

FM fixation systems consistently demonstrated lower deformation values than comparable LN configurations. FM systems with 0.8 mm thickness (tests 9 and 10) showed deformation values of 0.37631 mm and 0.35677 mm, respectively, with global stress values close to 360 MPa and plate stress values below 305 MPa.

The FM system with reinforcement and total inferior mesh extension (test 11) demonstrated a further reduction in deformation to 0.25795 mm, while maintaining plate stress at 300.37 MPa.

***Effect of plate thickness (0.8 mm vs. 1.0 mm)***

All fixation systems using 1.0 mm-thick plates (tests 12–15) exhibited deformation values below 0.56 mm. Dual reinforced LN plates with 1.0 mm thickness (test 12) showed a deformation of 0.56032 mm, while combined reinforced LN configurations with partial and total mesh extensions (tests 13 and 14) reduced deformation to 0.50217 mm and 0.26889 mm, respectively.

The FM reinforced system with total inferior mesh extension and 1.0 mm thickness (test 15) demonstrated the lowest deformation of all configurations, with a value of 0.25263 mm, and the lowest equivalent plate stress (246.72 MPa) among all tested models.

**Figure 2:**
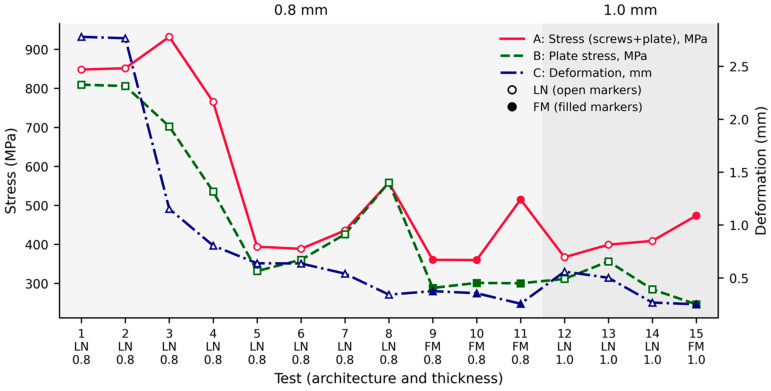
Mechanical behavior of mandibular fixation systems following 10 mm advancement. Finite element analysis results of 15 mandibular fixation configurations subjected to a vertical load applied to the lower central incisor. The x-axis represents the test number, with additional labeling indicating fixation architecture (LN or FM) and plate thickness ((0.8 mm in light gray and 1.0 mm in dark gray)). Curves represent: A, global equivalent stress considering screws and plate (MPa); B, equivalent stress in the plate (MPa); and C, global deformation of the fixation system (mm). Open markers correspond to LN (independent plate) configurations, while filled markers correspond to FM (integrated fixation) systems. Light gray background indicates tests performed with 0.8 mm-thick plates (tests 1–11), and darker gray background indicates tests using 1.0 mm-thick plates (tests 12–15).

Overall, the results demonstrated a progressive reduction in system deformation with increasing fixation complexity, incorporation of reinforcement and inferior mesh extensions, use of FM fixation architecture, and increased plate thickness (Figure 2). The simplest fixation systems exhibited the highest stress concentrations and deformation, whereas reinforced and integrated systems showed improved mechanical stability under the simulated loading conditions.

When compared with the simplest fixation system, represented by a single non-reinforced LN plate with a deformation of approximately 2.77 mm, reinforced and integrated configurations demonstrated a pronounced reduction in deformation. Reinforced LN plates reduced deformation to values close to 0.8 mm, dual LN configurations to approximately 0.64 mm, and FM systems to values below 0.38 mm, with the most stable configuration achieving a deformation reduction greater than 90%. Regarding plate thickness, fixation systems using 1.0 mm plates consistently exhibited lower equivalent stress values than their 0.8 mm counterparts, while deformation remained similarly low in reinforced and integrated configurations, indicating that increased plate thickness primarily improved stress distribution rather than producing additional reductions in deformation.

## 4. Discussion

Large mandibular advancements are used in a variety of clinical conditions. From cranio-mandibular malformations to obstructive sleep apnea, these conditions can be effectively treated through mandibular advancement techniques. Several approaches have been proposed, among which distraction osteogenesis represents a viable option. Its advantages include progressive advancement that allows movements exceeding 10 mm and the simultaneous expansion of surrounding soft tissues [12]. However, the use of more complex fixation systems, higher costs, and the need for significant patient compliance represent notable disadvantages of this method. In contrast, the use of plates and screws offers important benefits, including a single-stage procedure, lower risk, reduced costs, and simpler clinical implementation [13].

Finite element analysis was selected as the methodological approach for this study due to its capacity to isolate mechanical variables and evaluate stress distribution and deformation under controlled and reproducible conditions. This technique has been widely applied to investigate fixation stability after sagittal split ramus osteotomy and mandibular advancement, allowing systematic comparison of fixation designs under standardized loading conditions [4,11]. In the context of large mandibular advancements, finite element analysis provides valuable insight into early mechanical behavior, which is a critical phase for postoperative stability. In the present model, a 10 mm gap was created to assess the performance and stability of different fixation configurations. Although this computational model does not replicate a sagittal split ramus osteotomy, it provides a valid framework for evaluating the biomechanical effectiveness of fixation systems.

Alternative methodologies, such as in vitro mechanical testing using synthetic or cadaveric mandibles, have also been employed to evaluate fixation performance [11,14]. Although these approaches allow physical validation, they are limited by material variability and reduced reproducibility across multiple configurations. Clinical studies, while essential for outcome validation, are influenced by biological healing and patient-related factors, making them less suitable for isolated mechanical comparison of fixation designs. Consequently, finite element analysis remains a complementary and appropriate tool for preliminary mechanical assessment and design optimization [3].

The present finite element analysis demonstrated that plate design, plate thickness, and fixation architecture influenced the mechanical behavior of mandibular advancement fixation systems. Under a standardized 10 mm gap in mandibular osteotomy and vertical incisal loading, distinct patterns of stress distribution and deformation were observed among the evaluated configurations. Similar variability in mechanical response related to fixation design has been reported in previous finite element studies assessing mandibular advancement stability [14,15]. In terms of physiological bite force in healthy dentate adults, this typically ranges between 150 and 250 N, whereas maximum voluntary masticatory force in the molar region has been reported to range from 500 to 800 N, with mean values close to 600–700 N depending on sex and age [16,17]. In addition, under functional loading conditions, finite element analysis models indicate that equivalent stress values in the mandibular bone generally remain below 120–130 MPa, primarily concentrated in the condylar region and along the inferior border of the mandibular body. By contrast, during maximum voluntary biting, a marked increase in bone stress has been reported, with equivalent stress values reaching approximately 150–300 MPa [18,19]. Considering these clinical conditions, the use of a stronger configuration or reinforced plate could be an option in cases with a 10 mm or more gap between segments.

Simple straight plate configurations with reduced thickness exhibited the highest deformation values and increased stress concentration, indicating limited resistance to bending under functional loading. This behavior is consistent with prior biomechanical investigations demonstrating reduced stability of simpler fixation strategies in large mandibular advancements [1,11]. Large advancements have repeatedly been described as mechanically demanding scenarios, where insufficient fixation rigidity may compromise early postoperative stability [3].

The progressive reinforcement of plate design, including the incorporation of inferior mesh extensions, resulted in a marked reduction in system deformation and a more favorable distribution of stresses within fixation components. Comparable trends have been reported in previous finite element analyses evaluating modified plate geometries, where reinforced designs improved load-sharing capacity and reduced peak stress values [4,15]. These findings support the rationale for exploring alternative designs aimed at enhancing stability without increasing the number of fixation elements.

The comparison between independent plate fixation systems and integrated fixation architectures revealed that integrated systems generally exhibited lower deformation and reduced plate stress. This observation aligns with biomechanical reports suggesting that connected or frame-based fixation systems enhance mechanical coupling between fixation points, improving resistance to bending and rotational forces [20,21]. Importantly, these advantages were achieved using standard fixation systems rather than patient-specific implants.

Plate thickness also played a relevant role in fixation performance. Configurations using thicker plates demonstrated reduced deformation compared with thinner plates, particularly when combined with reinforced designs or integrated architectures. Similar findings have been reported in previous biomechanical studies showing that increased plate thickness contributes to enhanced stiffness and load-bearing capacity in mechanically demanding situations [22,23].

Several limitations of this study should be acknowledged. The analysis was restricted to a single loading condition and did not account for dynamic masticatory forces, muscle activity, or biological processes such as bone healing and remodeling. Material properties were assumed to be homogeneous and isotropic, patient-specific anatomical variability was not incorporated and the virtual models are not a full example of a clinically regular sagittal mandibular split osteotomy. These limitations are inherent to finite element modeling and have been similarly described in previous biomechanical studies of mandibular fixation [4,11]. However, the aim to establish the mechanical performance of different fixation configurations was obtained, and when isolated from other clinical variables can aid the clinical decision in the operating room.

The model used does not account for dynamic masticatory loads or muscle activity, nor does it incorporate individual anatomical variability related to age, sex, occlusal type, or craniofacial morphology. Consequently, the data obtained cannot be considered universal and should be interpreted as deriving from an averaged model based on standardized parameters. Nevertheless, our results allow the establishment of comparative criteria for the selection of fixation systems, whereby the choice of the fixation system should be tailored to the specific clinical scenario, considering the magnitude of mandibular advancement, the expected biomechanical conditions, and the requirements for primary stability.

The reported stress values were used solely for relative comparisons among the different fixation configurations, assessing the effects of plate design, thickness, and system architecture on stress distribution and deformation. They were not directly compared to material performance or fatigue limits, as the study focused on characterizing comparative mechanical behavior under a standardized vertical load applied to the central incisor. This approach allows identification of the configurations with greater mechanical stability without directly extrapolating clinical failure predictions.

From a clinical perspective, the present findings suggest that meaningful improvements in mechanical stability can be achieved through optimization of standard fixation systems. Reinforced plate designs, increased plate thickness, and integrated fixation architectures may offer improved resistance to deformation, which could be particularly beneficial in large mandibular advancements.

Importantly, these improvements were observed using standard fixation systems that remain widely accessible and cost-effective. In clinical settings where patient-specific implants are not available or economically feasible, careful selection and optimization of standard straight plate configurations may represent a practical strategy to enhance fixation stability. The biomechanical evidence provided by this study may assist surgeons in tailoring fixation strategies according to the mechanical demands of mandibular advancement procedures.

This article presents a structural, mechanical, and mathematical evaluation of osteosynthesis plate performance in mandibular osteotomy. Such analysis is critical for determining the most appropriate fixation strategy, with direct relevance to clinical decision-making. The study identifies which osteosynthesis configuration provides greater mechanical reliability in demanding mandibular advancement conditions, where rigid internal fixation is essential to maintain short- and mid-term stability. The findings offer practical, evidence-based guidance for surgeons when selecting fixation systems in challenging advancement procedures.

## 5. Conclusions

Plate design, plate thickness, and fixation architecture influenced the mechanical stability of mandibular advancement in this finite element analysis. Reinforced plate designs and increased thickness reduced deformation and improved stress distribution. These results highlight that reinforced fixation configurations with an integrated architecture provide the most favorable combination of stress reduction and deformation control.

The selection of the fixation method should be based on the specific clinical scenario. Situations involving higher biomechanical demands may require fixation strategies providing greater rigidity, whereas less demanding cases may be managed with simpler configurations without compromising mechanical stability.

## Figures and Tables

**Table 1:** Finite element analysis results of mandibular fixation systems following 10 mm advancement. Summary of mechanical outcomes obtained from 15 finite element simulations evaluating different mandibular fixation configurations after a 10 mm mandibular advancement. Fixation systems varied according to plate design, number of plates, fixation architecture (LN or FM), and plate thickness (0.8 mm or 1.0 mm). Outcome measures include: A, global equivalent stress considering both screws and plate (MPa); B, equivalent stress in the plate (MPa); and C, global deformation of the fixation system (mm) under a vertical load applied to the lower central incisor.

Test	Fixation (Design)	A. Global Equivalent Stress Including Screws and Plate	B. Equivalent Stress in the Plate (MPa)	C. Global Deformation of the Fixation System (mm)	Model
1	1 plate LN simple 0.8	848.2	809.38	2.7773	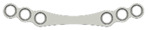
2	1 plate LN reinforced 0.8	851.51	805.88	2.7643	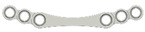
3	1 plate LN reinforced 0.8 (partial mesh)	931.98	702.26	1.1526	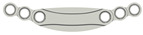
4	1 plate LN reinforced 0.8 (full mesh)	765.23	535.51	0.80527	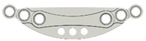
5	2 plates LN simple 0.8	394.06	331.6	0.63842	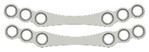
6	2 plates reinforced 0.8	388.45	360.37	0.63641	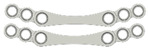
7	1 plate LN simple 0.8 + 1 plate LN reinforced 0.8 (partial mesh)	435.47	425.82	0.54105	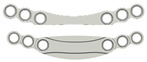
8	1 plate LN simple 0.8 + 1 plate LN reinforced 0.8 (full mesh)	558.33	558.33	0.34384	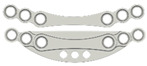
9	1 plate FM simple 0.8	360.24	288.44	0.37631	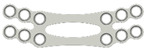
10	1 plate FM reinforced 0.8	359.78	301.33	0.35677	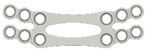
11	1 plate FM reinforced 0.8 (full mesh)	514.98	300.37	0.25795	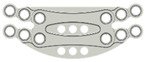
12	2 plate LN reinforced 1.0	367.04	311.42	0.56032	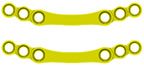
13	1 plate LN reinforced 1.0 + 1 plate LN reinforced 1.0 (partial mesh)	399.12	356.29	0.50217	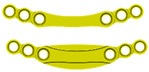
14	1 plate LN reinforced 1.0 + 1 plate LN reinforced 1.0 (full mesh)	408.8	284.83	0.26889	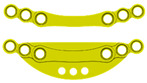
15	1 plate FM reinforced 1.0 (full mesh)	473.55	246.72	0.25263	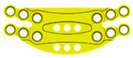

## Data Availability

The original contributions presented in this study are included in the article. Further inquiries can be directed to the corresponding author.
